# Tobacco Control in State Comprehensive Cancer Control Plans: Opportunities for Decreasing Tobacco-Related Disease

**Published:** 2007-06-15

**Authors:** Jerold R Mande, Briseis A Kilfoy, Karen Suchanek Hudmon

**Affiliations:** Yale Cancer Center, Yale University School of Medicine; Department of Epidemiology and Public Health, Yale University School of Medicine, New Haven, CT; Department of Pharmacy Practice, Purdue University School of Pharmacy and Pharmaceutical Sciences, West Lafayette, IN

## Abstract

**Introduction:**

Comprehensive cancer control plans published by state, tribal, and territorial health agencies present an excellent opportunity to help prevent tobacco-related and other cancers. In this analysis, we sought to estimate the extent to which tobacco control activities outlined in state comprehensive cancer control plans incorporated the tobacco control recommendations presented by the Centers for Disease Control and Prevention (CDC) in *Best Practices for Comprehensive Tobacco Control Programs — August 1999* (*Best Practices*) and *The Guide to Community Preventive Services: Tobacco Use Prevention and Control* (*The Guide*).

**Methods:**

We analyzed the 39 available state comprehensive cancer control plans to determine which of the CDC tobacco control recommendations were incorporated. We then summarized these data across the 39 states.

**Results:**

The 39 states incorporated a mean of 5.6 recommendations from *Best Practices *(SD, 2.8; range, 0–9) and 3.9 recommendations from *The Guide* (SD, 1.9; range, 0–6). Nearly one-half of state plans (48.7%) addressed funding for tobacco control; of these, 52.6% (25.6% of total) delineated a specific, measurable goal for funding.

**Conclusion:**

The extent to which tobacco control is addressed in state comprehensive cancer control plans varies widely. Our analysis revealed opportunities for states to improve compliance with CDC's tobacco-related recommendations for cancer control.

## Introduction

Tobacco use, the leading known preventable cause of death in the United States (1), results in an estimated 20% of all deaths annually (2). Smoking contributes to approximately 30% of all cancers in the developed world and is responsible for an estimated 90% of lung cancers and for many other malignancies (3). Because tobacco use is a modifiable risk factor, tobacco control offers enormous opportunity for reducing tobacco-attributable morbidity and mortality, not only from cancer but also from cardiovascular disease, pulmonary disease, and a wide range of other conditions.

Studies show that comprehensive approaches to tobacco control, including approaches that focus on educational, economic, clinical, and regulatory strategies, are particularly effective in reducing the prevalence of tobacco use and associated disease and disability (4,5). Tobacco control has effectively reduced the prevalence of smoking in several states, including California, Massachusetts, Oregon, and Florida (6), and evidence gathered in these states and elsewhere has led to the development of two tobacco control guidelines by the Centers for Disease Control and Prevention (CDC): *Best Practices for Comprehensive Tobacco Control Programs* *—August 1999 *(7) (*Best Practices*) and *The Guide to* *Community Preventive Services: Tobacco Use Prevention and Control* (8) (*The Guide*). Both reports offer critical reviews of the literature and identify approaches to tobacco control supported by the existing body of scientific evidence (9). The products of decades of research, these guidelines are intended to inform policy decisions so that states are able to employ the most effective measures of tobacco use prevention, tobacco cessation, and chronic disease treatment.

To enhance cancer control efforts, CDC currently supports the National Comprehensive Cancer Control Program (NCCCP), which provides financial support and expert advice to aid states in developing cancer control plans. Comprehensive cancer control is based on the premise that effective cancer control planning and program implementation at the local, state, and national levels address a continuum of services, beginning with primary prevention and early detection and progressing through quality cancer treatment and addressing the needs of cancer survivors (10).

With approximately $15 million in Congressional appropriations in fiscal year 2005, CDC supported efforts to build coordinated and focused cancer control programs in all states, the District of Columbia, six tribal jurisdictions, and six Pacific Island territories (10). By August 2005, 39 states had published comprehensive cancer control plans, and 63 states, tribal, and territorial health agencies had obtained funding for their programs. CDC specifically recommends that states base their comprehensive tobacco control plans on the evidence-based strategies delineated in *Best Practices* and *The Guide*. Although the number of states that have developed a comprehensive cancer control plan has increased in recent years, and nearly all of these plans include a tobacco control section, no study has assessed the extent to which these sections are congruent with CDC's tobacco control guidelines.

Our goal was to estimate the extent to which the tobacco control sections of state comprehensive cancer control plans incorporate the tobacco control recommendations in *Best Practices* and *The Guide*, so that missed opportunities can be brought to the attention of stakeholders and addressed in future revisions to these plans. We also assessed whether each state plan addressed the funding objectives for comprehensive tobacco control activities recommended in *Best Practices*.

## Methods

### Sources of data

Our analysis focused only on the 50 states, omitting the District of Columbia, tribal jurisdictions, and Pacific Island territories. During summer 2005, we obtained the most recent editions of the available comprehensive cancer control plans by contacting an administrator of each state planning group and by searching for published versions of the plans on the Internet, particularly on the NCCCP Web page (http://www.cdc.gov/cancer/ncccp/index.htm). We extracted the tobacco-related content from each plan and asked the planning group administrators to review their state's information to confirm that all goals, strategies, and objectives regarding tobacco control were accurate and complete. Eleven states had not published cancer control plans as of August 2005 and were not included in the analysis.

### Analysis

We analyzed the 39 available state comprehensive cancer control plans to determine which of the CDC tobacco control recommendations were incorporated. We then summarized these data across the 39 states.

To analyze whether state plans addressed the tobacco control recommendations in *Best Practices* and *The Guide*, we used dichotomous indicators: yes, the recommendation is addressed in the plan, or no, the recommendation is not addressed in the plan. We also used dichotomous indicators to assess whether the plans specifically addressed funding levels: yes, tobacco control funding is addressed in the plan, or no, tobacco control funding is not addressed in the plan. For example, because Delaware's plan asserts that the state will "at a minimum, fund comprehensive statewide tobacco-control activities at $8.6 million (the CDC-recommended minimum)" (13), we classified the plan as having addressed funding.

## Results

**Figure 1:**
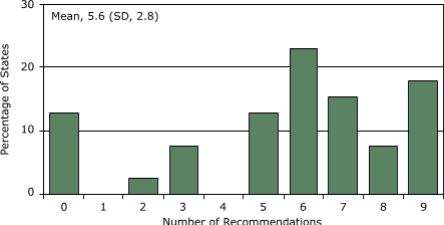
Extent (in percentage) to which state comprehensive cancer control plans (N = 39) incorporate recommendations (N = 9) from *Best Practices for Comprehensive Tobacco Control Programs* (7), by number of recommendations included, United States, 2005.

**Figure 2:**
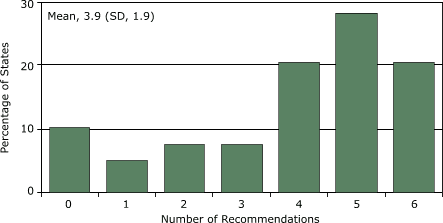
Extent (in percentage) to which state comprehensive cancer control plans (N = 39) incorporate recommendations (N = 6) from *The Guide to Community Preventive Services: Tobacco Use Prevention and Control* (8), by number of recommendations included, United States, 2005.

The state comprehensive cancer control plans addressed a mean of 5.6 of the 9 recommendations from *Best Practices* (SD, 2.8; range, 0–9) (Figure 1), with 7 states integrating all of the recommendations and 5 integrating none. The state plans addressed a mean of 3.9 of the 6 recommendations from *The Guide *(SD, 1.9; range 0–6) (Figure 2), with 8 states integrating all of the recommendations and 4 integrating none.

The recommendation on cessation programs was the most commonly addressed (86.8%); the recommendation on chronic disease programs was the least commonly addressed (34.2%) (Table). Nearly one half of state plans (48.7%) addressed funding for tobacco control; of these, 52.6% (25.6% of total) included a specific, measurable goal, either a dollar amount or a target percentage of CDC's minimum funding recommendation.

**Table:** 

**Number of Recommendations Included**	**Percentage of States Including This Number**
0	12.8
1	0
2	2.6
3	7.7
4	0
5	12.8
6	23.1
7	15.4
8	7.7
9	17.9

**Table:** 

**Number of Recommendations Included**	**Percentage of States Including This Number**
0	10.3
1	5.1
2	7.7
3	7.7
4	20.5
5	28.2
6	20.5

## Discussion

Tobacco control should be a prominent element in all comprehensive cancer control plans. Our content analysis, however, demonstrates wide variation in the extent to which state cancer control plans incorporate CDC's recommended tobacco control measures. Only one in four state plans delineated specific, measurable funding objectives. States that do not effectively plan and address tobacco control in their comprehensive cancer control plans might be losing an important opportunity to improve public health (14).

A strength of our study is that all states included in the analysis had the opportunity to confirm the tobacco control content that we extracted from their larger comprehensive cancer control plan. Several factors may influence the extent to which state plans address individual tobacco control recommendations, including political resistance or efforts by the tobacco industry to derail recommendations such as the implementation of workplace smoking bans or increases in tobacco sales taxes. Some plans might have been in existence long enough to benefit from evaluation and revision, whereas plans developed more recently may not yet have been evaluated. Some states might have tobacco control components in a tobacco control plan but might not have integrated this information into their comprehensive cancer control plan. Finally, the strategies, goals, and objectives addressed in comprehensive cancer control plans might not accurately reflect the actual tobacco control activities at the state level.

The inability of dichotomized variables to reveal the extent of variation among states' responses to each recommendation limits our content analysis. For example, in response to the recommendation to increase the tax on cigarettes, some states have raised the cost per pack by $1.00 or more, whereas other states have increased the cost by less than $0.25. Another limitation of our analysis is that it did not reveal the relative importance or effectiveness of individual recommendations. Finally, despite our careful content analysis of the state plans, our characterization of whether a state addressed the various recommendations is subject to interpretation.

The National Cancer Institute's (NCI) 2015 Challenge Goal emphasizes the importance of careful planning in efforts to meet the goal of "eliminating the suffering and death due to cancer" (15). The goals for comprehensive cancer control plans are based on a series of objectives (enhance infrastructure, mobilize support, utilize data and research, build partnerships, assess and address cancer burden, and conduct evaluations) and desired outcomes to help states develop the most effective plan in light of their needs and resources (8). Groups of stakeholders from multiple segments of the community develop these plans, and multiple organizations have a sense of ownership. Although in many instances a state department of health has served as a catalyst for this process and accepts funding from CDC to support planning and implementation, in nearly every case a partnership or coalition of many agencies, groups, and individuals is responsible for identifying the priorities described in the plan and the activities to be implemented (14). Neither *Best Practices* nor *The Guide* ranks its recommendations by importance; consequently, singling out a recommendation that might be most important for a given state is not possible.

As of November 2006, 49 states had published comprehensive cancer control plans, an increase that represents tremendous growth since 1998, when the first 6 states received funding to develop plans. If these plans are, however, to enable the nation to reach such cancer prevention goals as NCI's 2015 Challenge Goal, they must employ evidence-based strategies to control cancer such as those provided in *Best Practices* and *The Guide*.

Our analysis indicates room for improvement in the extent to which most state plans address tobacco use. Policy makers and planners should examine their state's comprehensive cancer control plan and their tobacco control plan, if one exists, and achieve concordance between these plans and with each CDC recommendation. Each plan should address funding levels, since sufficient funding is essential for progression from program planning to implementation. Our results call the states to action, warranting 1) careful creation or modification of state comprehensive cancer control plans to adhere to the recommendations set forth in *Best Practices* and *The Guide* and 2) sufficient funding to support full enactment of these plans.

## Figures and Tables

**Table:** Percentage of State Comprehensive Cancer Control Plans That Incorporate Tobacco Control Recommendations From *Best Practices for Comprehensive Tobacco Control Programs* (7) and *The Guide to Community Preventive Services: Tobacco Use Prevention and Control* (8), United States, 2005

Recommendations	Plans Addressing Recommendations, **%**
In ***Best Practices for Comprehensive Tobacco Control Programs***	
Community programs to reduce tobacco use	76.3
Chronic disease programs to reduce tobacco-related disease	34.2
School programs	68.4
Enforcement	55.3
Statewide programs	84.2
Counter-marketing	63.2
Cessation programs	86.8
Surveillance and evaluation	52.6
Administration and management	39.5
In ***The Guide to Community Preventive Services: Tobacco Use Prevention and Control***	
Smoking bans and restrictions	84.2
Increase in the unit price for tobacco products	47.4
Media campaigns and interventions	60.5
Provider reminder systems and provider education	71.1
Reduction of patient out-of-pocket costs for treatment	71.1
Patient telephone support (quitlines)	50.0
